# Ancient DNA from latrines in Northern Europe and the Middle East (500 BC–1700 AD) reveals past parasites and diet

**DOI:** 10.1371/journal.pone.0195481

**Published:** 2018-04-25

**Authors:** Martin Jensen Søe, Peter Nejsum, Frederik Valeur Seersholm, Brian Lund Fredensborg, Ruben Habraken, Kirstine Haase, Mette Marie Hald, Rikke Simonsen, Flemming Højlund, Louise Blanke, Inga Merkyte, Eske Willerslev, Christian Moliin Outzen Kapel

**Affiliations:** 1 Department of Plant and Environmental Sciences, University of Copenhagen, Frederiksberg, Denmark; 2 Centre for GeoGenetics, Natural History Museum of Denmark, University of Copenhagen, Copenhagen K, Denmark; 3 Department of Veterinary Disease Biology, University of Copenhagen, Frederiksberg, Denmark; 4 BioArchaeological Research Bureau, Den Haag, The Netherlands; 5 Odense Bys Museer, Odense, Denmark; 6 Centre for Urban Network Evolutions, School of Culture and Society, Aarhus University, Højbjerg, Denmark; 7 Environmental Archaeology and Materials Science, National Museum of Denmark, Kgs. Lyngby, Denmark; 8 Museum of Copenhagen, Copenhagen V, Denmark; 9 Moesgaard Museum, Højbjerg, Denmark; 10 Department of Archaeology, School of Culture and Society, Aarhus University, Aarhus, Denmark; 11 The Saxo Institute, University of Copenhagen, Copenhagen S, Denmark; 12 Department of Zoology, University of Cambridge, Downing St, Cambridge, United Kingdom; 13 Sanger Institute, Hinxton, Cambridge, United Kingdom; Seoul National University College of Medicine, REPUBLIC OF KOREA

## Abstract

High-resolution insight into parasitic infections and diet of past populations in Northern Europe and the Middle East (500 BC- 1700 AD) was obtained by pre-concentration of parasite eggs from ancient latrines and deposits followed by shotgun sequencing of DNA. Complementary profiling of parasite, vertebrate and plant DNA proved highly informative in the study of ancient health, human-animal interactions as well as animal and plant dietary components. Most prominent were finding of soil-borne parasites transmitted directly between humans, but also meat-borne parasites that require consumption of raw or undercooked fish and pork. The detection of parasites for which sheep, horse, dog, pig, and rodents serves as definitive hosts are clear markers of domestic and synanthropic animals living in closer proximity of the respective sites. Finally, the reconstruction of full mitochondrial parasite genomes from whipworm (*Ascaris lumbricoides*) and roundworm species (*Trichuris trichiura* and *Trichuris muris*) and estimates of haplotype frequencies elucidates the genetic diversity and provides insights into epidemiology and parasite biology.

## Introduction

Human infections with intestinal worms were widespread in Europe until the middle of the last century, but are today primarily confined to rural communities in developing countries with low levels of sanitation, insufficient water refinement, and where humans and animals live in close proximity. Under such settings, the giant roundworm *Ascaris lumbricoides* and the human whipworm *Trichuris trichiura* are still found at high prevalence. These two soil-transmitted parasites have been widely identified in archaeological samples associated with human remains and coprolites in areas where they are no longer endemic [1].

Traditional palaeoparasitological methods rely on morphological examinations of recovered parasite eggs. While a few species may reliably be identified using microscopy, others can only be identified to genus level due to overlapping morphological characteristics of the eggs of closely related species [2,3]. This prevents further elucidation of specific host-parasite associations. Contrary, DNA based analysis may identify parasites at the species level and, as most parasites are host specific with distinct life cycles, they can serve as markers for the presence of their intermediate and/or definitive hosts. This is particularly the case for the *Trichuris* genus, which comprises more than 10 distinct species with narrow host specificity where e.g. *T*. *trichiura* infects humans, *T*. *suis* infects pigs and *T*. *muris* infects mice [4–7]. *Trichuris* spp., as well as *Ascaris* spp., exhibits direct life cycles where worms residing in the host intestine, excrete eggs to the environment with host faeces. Transmission to a new host occurs through ingestion of infective eggs from the environment or through contaminated foods. High host specificity of *T*. *trichiura* along with the global distribution and wide detection of eggs in archaeological samples makes this parasite a suitable indicator of human migrations [8]. This is supported by a recent study on populations of modern *T*. *trichiura* isolates, which suggested that worms were distributed with human migration first from Africa to Asia and then further to South America whereas *T*. *suis* might have been distributed through a combination of pig migration and transport of domesticated pigs,—and their parasites [9]. *Ascaris* in humans (*A*. *lumbricoides*) and pigs (*A*. *suum*) are very closely related and cannot be distinguished based solely on mitochondrial DNA markers [10]. However, as *Ascaris* and *Trichuris* most often are co-detected in latrine samples and human intestinal contents from Europe and the Middle East [11–15], we use the term *A*. *lumbricoides* to describe *Ascaris* based on DNA findings when co-identified with *T*. *trichiura*.

Identifying ancient parasites, which exhibit indirect life cycles involving an animal as intermediate host and humans as definitive hosts, may reveal our ancestors’ dietary preferences and animal exploitation history. Tapeworms, such as *Taenia solium* and *T*. *saginata*, exemplify this, as tissue cysts are transmitted to humans by consumption of undercooked pork or beef, respectively. Notably, in ancient Greenlandic kitchen middens only the tapeworm species *T*. *hydatigena*, *T*. *multiceps* and *E*. *canadensis* were identified by DNA analysis, inferring the presence of canid and ungulate hosts, typically sheep and reindeer, and thereby indicating human resource economy [16].

Currently, the implementation of DNA based analysis in palaeoparasitology is limited to first-generation sequencing of PCR amplicons [17–19], a next-generation barcoding approach for identifying 8 genera of helminths [20] and our recent publication on the resource economy in ancient Greenland [16]. Here we extend the shotgun DNA sequencing analysis on ancient parasite eggs; identify parasite infections to the species level, use them as markers of diet and specifically as markers for which animals who lived in the immediate proximity of the sample point. We identify vertebrate DNA, for which the majority of taxa are domesticated animals, typically livestock, and animals associated with hunting or fishing practices. Diet is further addressed through plant DNA with focus on edible plants. Finally, shotgun sequencing has allowed us to reconstruct multiple ancient mitochondrial genomes of *A*. *lumbricoides*, *T*. *trichiura* and *T*. *muris*, analyse their phylogenies and estimate the number of haplotypes present in a sample. The approach shows obvious potential for future reconstructions of nuclear genomes from ancient parasites.

## Methods

### Samples and egg extraction

**Fig 1:**
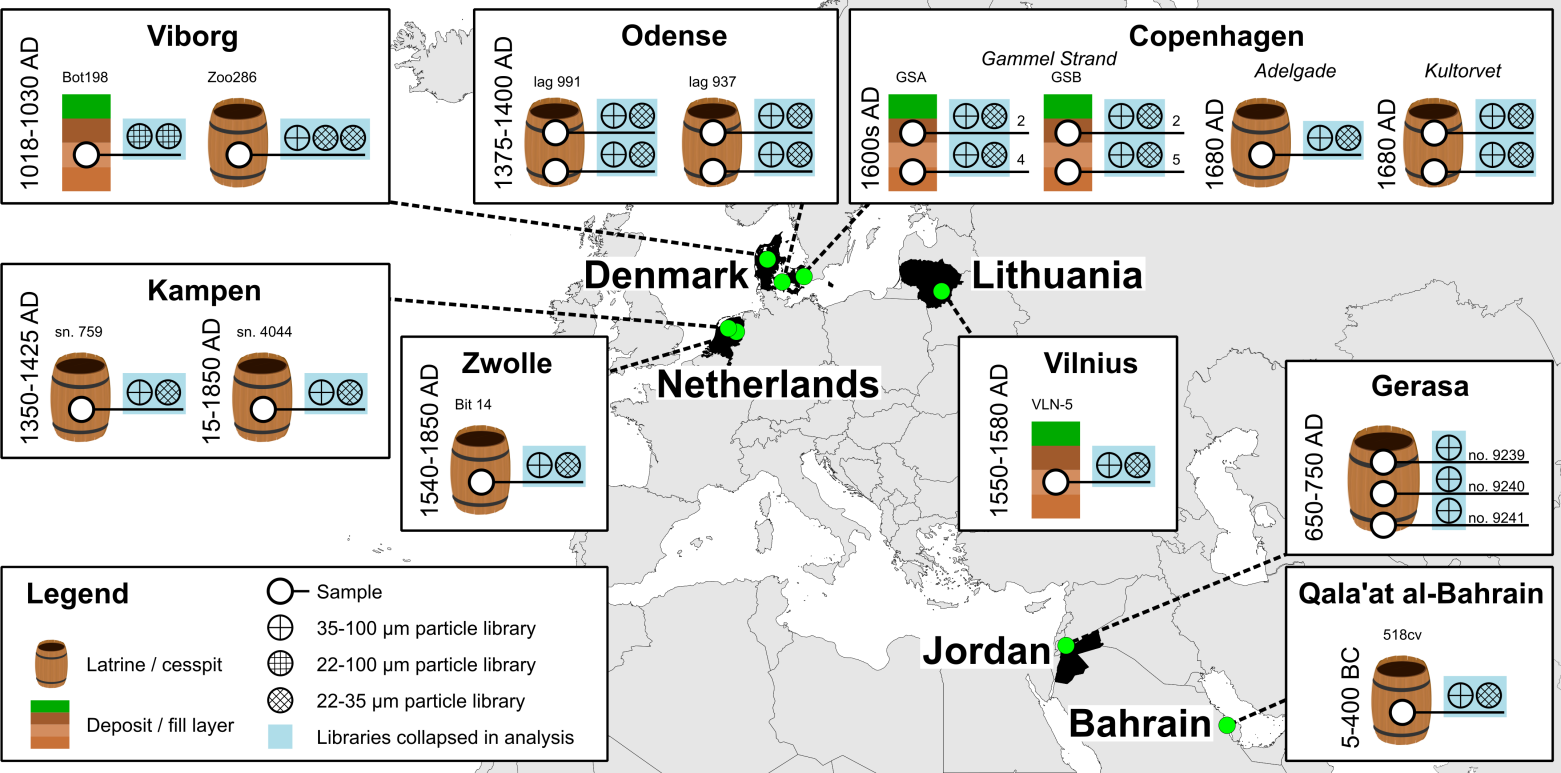
Sampling locations for archaeologically classified sample types. Samples were processed for particle size selection by wet sieving on filters of designated sizes. Individual DNA libraries were prepared for the illustrated combinations of sample and particle sizes, then sequenced and collapsed to sample level for further analysis. The 22–35 μm particle isolates of the Jordan, Gerasa samples were devoid of any particles and not processed for sequencing.

Samples were collected from archaeological excavations of human latrines and deposits with high levels of organic material. Initially, samples were screened for the presence of *Ascaris* spp. or *Trichuris* spp. eggs to identify human fecal material, by dissolving 10 gram of sample in 50 mL tubes filled with flotation buffer (FB) (glucose monohydrate 375 g/L + sodium chloride 250 g/L). Tubes were centrifuged (15–100 g, 5 min) and the top 1 mL of supernatant was transferred to McMaster counting chambers and examined with a microscope at 100/400x magnification. Samples chosen for further processing are illustrated in Fig 1, while specimen numbers are listed in S1 Table. No permits were required for the described study, which complied with all relevant regulations. The Danish samples were taken from excavations carried out within the Danish Museum Act, which includes the undertaking of scientific sampling and analysis by non-written agreement with the local archaeologists. Permit for analysis of the Jordanian samples is included in the export permit of the samples by the Amman and Jarash offices of the Department of Antiquities. The Dutch and Lithuanian samples were analysed at the discretion of the local archaeologists.

From each sample, 75–503 g (average 183 g) were processed by selecting particles based on flotation in high density liquid and size, as previously described [19]. In brief, samples were suspended in FB and centrifuged. The sediment was discarded after wet sieving the supernatant on stacked 100 and 22.4 μm filters. The material collected from the 22.4 μm filter was re-suspended in FB, centrifuged and supernatant wet sieved on stacked 35.5 and 22.4 μm filters. The samples were collected and termed ‘sample ID’ followed by ‘A’ for 35.5–100 μm particles and ‘B’ for 22.4–35.5 μm particles, unless otherwise specified. 10% of the sample was subjected to morphological examination and the remaining 90% for DNA analysis. Morphological examination was performed on microscope slides at 100x and 400x magnification and quantification of eggs performed either on fixed microscope slides or in McMaster counting chambers. The reported ‘number of eggs in sample’ was extrapolated from egg count and proportion of total analysed sample. All egg extractions were carried out in a dedicated palaeoparasitological laboratory at the Department of Plant and Environmental Sciences (PLEN), University of Copenhagen.

### DNA sequencing

Filtered samples, (0.02 g–0.58 g, average of 0.11 g) were processed for DNA extraction and library preparation in dedicated aDNA laboratories at the Centre for GeoGenetics (CGG), Natural History Museum, University of Copenhagen, in accordance with strict aDNA-specific requirements as previously described[19], although eluted in 60 μL elution buffer. Blunt end DNA libraries were prepared using NEBNext DNA Sample Prep Master Mix Set for 454 (E6070) and Illumina-specific adapters [21] following established protocols [22,23], hence without the ssDNA isolation module. The following exceptions to the protocols were made: intermittent reaction clean-ups were performed using the MinElute PCR purification kit (Qiagen), with an improved binding buffer that has proved highly efficient in recovering very short DNA fragments as described elsewhere [23]; adaptors were used at final concentration of 0.5 μM and the fill-in reaction was performed for 20 min at 60° C and inactivated for 20 min at 80°C. DNA libraries were amplified using a nested PCR approach in a first round reaction of 50 μL for 12 PCR cycles, 5 μL of product was used as template in the second reaction run for 10–16 cycles. PCR cycling and post-PCR handling was performed in modern DNA laboratories physically separated from the aDNA laboratories. Second round PCR’s were examined on a 2% agarose gel, purified using the MinElute kit following manufacturer’s instructions, then quantified on a Qubit 2.0 using the dsDNA HS kit (Thermo Fischer) and finally on a Bioanalyzer (Agilent Technologies) using the Agilent High Sensitivity DNA kit. For libraries not visible on the gel, or if the concentration after purification was <5 nM, the second round PCR was repeated with a higher number of cycles (up to 16). Purified libraries were pooled at concentrations of 5–20 nM before shotgun sequencing using 100-bp single read chemistry on a HiSeq 2000/2500 platform at The Danish National High-Throughput DNA Sequencing Centre. Negative controls were prepared for egg extraction, library preparation and PCR amplification steps and processed along with samples in the subsequent steps. Extraction controls were sequenced along with samples and processed together in subsequent analysis.

### Pre-processing sequence reads

Sequencing data was base called using Illumina software CASAVA 1.8.2 and sequences were de-multiplexed with a requirement of full match for the 6-nucleotide index that was used for library preparation. Sequences were trimmed using Adapter Removal v2 [24] to remove adapter sequences and stretches of mixed low quality bases and/or Ns from both the 5’ and 3’ end as well as discarding reads shorter than 30 nt. Sequence data was merged to library level and an additional filtering of low-quality reads was performed using the SGA–String Graph Assembler [25] *preprocess* command, option *dust-threshold* set to 3. Exact match read duplicates were removed, by first indexing the reads using *sga index* and then filtering using *sga filter* with the *no-kmer-check* disabled.

### Identifying helminth, vertebrate and plant DNA

The ‘lowest common ancestor’ (LCA) approach as described in detail elsewhere[16], was used to identify hits to mitochondrial and plastid genomes (https://github.com/frederikseersholm/getLCA). The mitochondrion reference database was acquired from NCBI (ftp://ftp.ncbi.nlm.nih.gov/refseq/release/mitochondrion/) and supplemented with *Trichuris* sp. sequences (KC461179, KT449822, KT449823, KT449826) and *Ascaris* sp. sequences (KY045800, KY45801, KY045804 and KY045805) to better encompass sequence variation in these genera. Similarly the plastid reference database was obtained from ftp://ftp.ncbi.nlm.nih.gov/refseq/release/plastid/. Taxonomic IDs were added to each fasta header in the databases using the NCBI file gi_taxid_nucl.dmp (ftp.ncbi.nih.gov/pub/taxonomy/gi_taxid_nucl.dmp.gz), names.dmp and nodes.dmp (ftp.ncbi.nih.gov/pub/taxonomy/taxdump.tar.gz), and custom scripts[16]. Sequences were mapped to the two databases using bowtie2 version 2.2.4 [26], reporting up to 500 alignments per read. The LCA algorithms (https://github.com/frederikseersholm/getLCA) were applied to the sam output files and the lowest common ancestor from the best hits for each read was returned, as in Seersholm et al (2016) [16]. Briefly, each read is assigned to the lowest common ancestor of the best hits to the database, based on edit distance. If a read has only a single best alignment the read is assigned directly to the taxa of that reference sequence. If the read has several equally good alignments (same edit distances), on the other hand, the read is assigned to the lowest common ancestor of the best hits, based on the NCBI taxonomy files names.dmp and nodes.dmp (ftp.ncbi.nih.gov/pub/taxonomy/taxdump.tar.gz). Reads identified as the best hit(s) were discarded if sequence identity to the reference was less than 95%.

**Fig 2:**
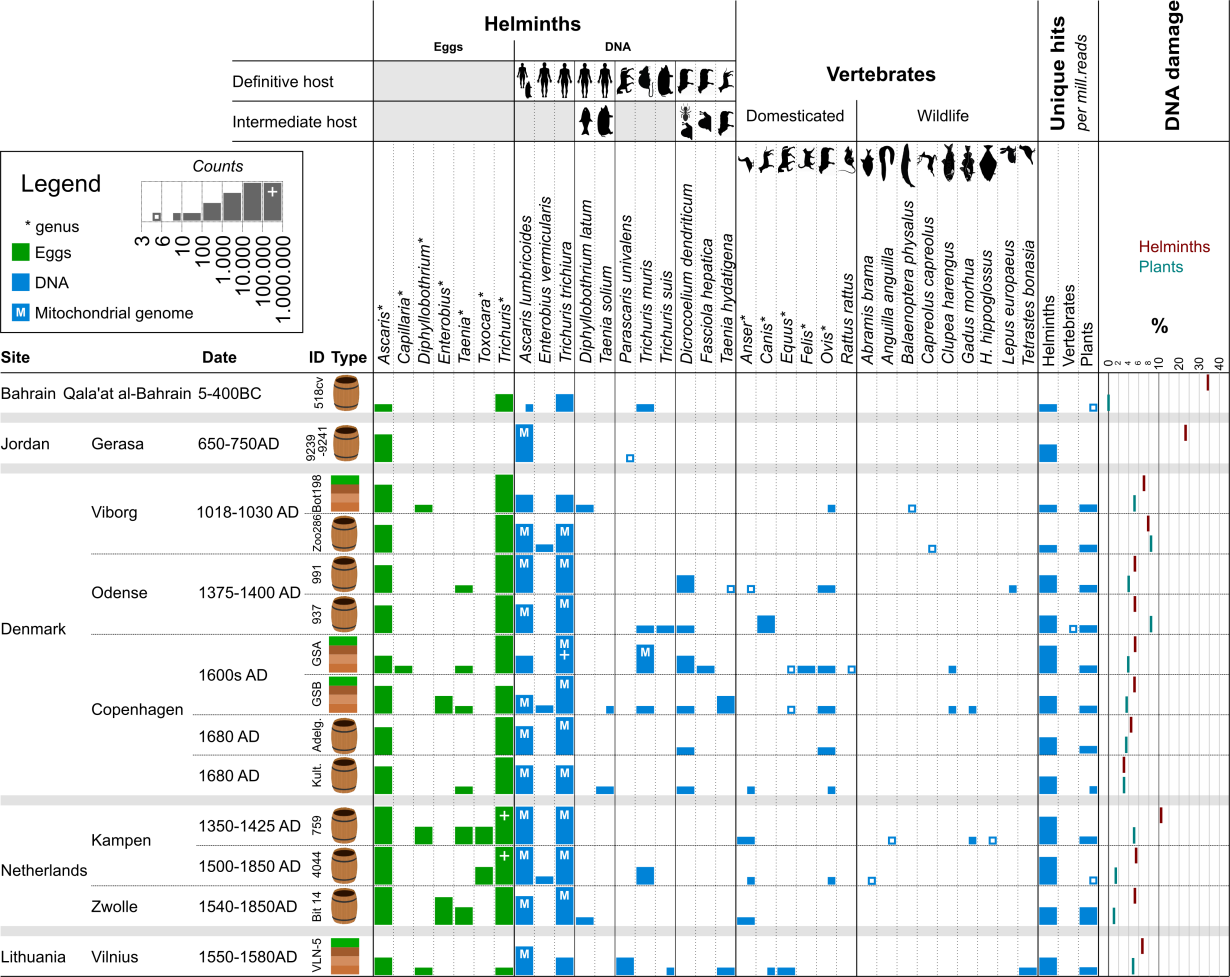
The most abundant taxa of helminths and vertebrates identified in the samples. Bars are logarithmic in size and show the estimated number of eggs in the sequenced sample and the number of DNA reads uniquely assigned to species and/or genus level. Helminths and vertebrates were identified based on sequence similarity to mitochondrial references, while plants were identified based on sequence similarity to plastid references. The typical hosts for the identified helminths are shown as the definitive host (*i*.*e*. the host where the parasite reproduces sexually and from which eggs are excreted) and intermediate hosts (*i*.*e*. host required for completion of the parasite life cycle). DNA damage is illustrated as the average frequency of C to T transition on the first 5’ position and the G to A transition on the first 3’ position of reads uniquely assigned to the helminth taxa presented here or the edible plant taxa presented in Fig 3.

Mitochondrial sequence hits are reported as ‘helminths’, which includes the ‘platyhelminthes’ and ‘nematoda’ phylum assignations, whereas ‘vertebrates’ includes ‘vertebrata’ sub-phylum assignations. Reads were mapped to sample level prior to reporting in Fig 2. The 11 most abundant helminth species of humans and animals are reported, while reads assigned to the *Ascaris* genus or the species, *A*. *lumbricoides or A*. *suum*, were reported as *A*. *lumbricoides*. The most abundant vertebrate taxa (species or genus) with more than 3 uniquely assigned reads are reported in Fig 2. Reads mapping to the following genus and species have been collapsed to genus level as follows: *Canis* contains *Canis*, *Canis lupus*, *Canis lupus familiaris* and *Canis lupus chanco*; *Equus* contains *Equus*, *Equus* (*ferus*) *przewalskii* and *Equus* (*ferus*) *caballus*; *Felis* contains *Felis*, *Felis silvestris*, *Felis catus* and *Felis margarita*; *Ovis* contains *Ovis* and *Ovis orientalis*.

The following taxa have been excluded from the reported vertebrate results: *Homo sapiens* and related primates; *Bos*, *Gallus* and *Sus* genera–observed many samples and DNA extraction controls and known to be common laboratory contaminants [27]; *Meleagris gallopavo* (Wild Turkey)–was observed in all DNA extraction controls and more so in highly degraded samples from the Middle East; *Pelodiscus sinesis* (Chinese Softshell Turtle), *Phelsuma guimbeaui* and *Phyllodactylus unctus* (both Geckos)–which were identified in many samples although with a maximum of 5 reads.

**Fig 3:**
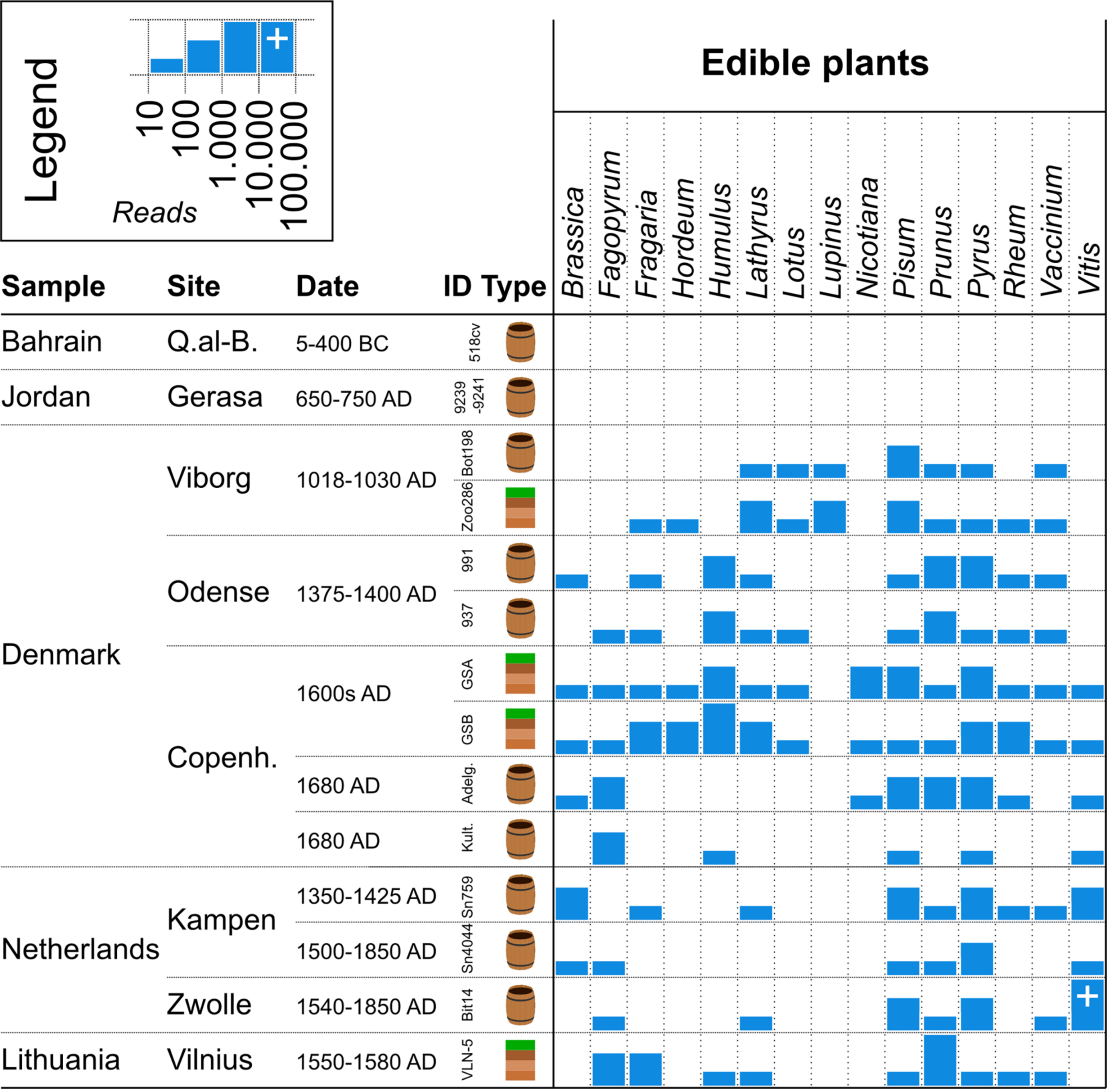
Edible plant identifications. The 15 most abundant plants, which are consumed by humans and identified in the samples are shown. Bars represent the number of uniquely assigned DNA reads, *i*.*e* assigned to species and/or genus level, then collapsed to genus level. Only assignments with more than 10 reads and only samples for which the combined reads exhibit ancient DNA damage profiles are shown.

**Fig 4:**
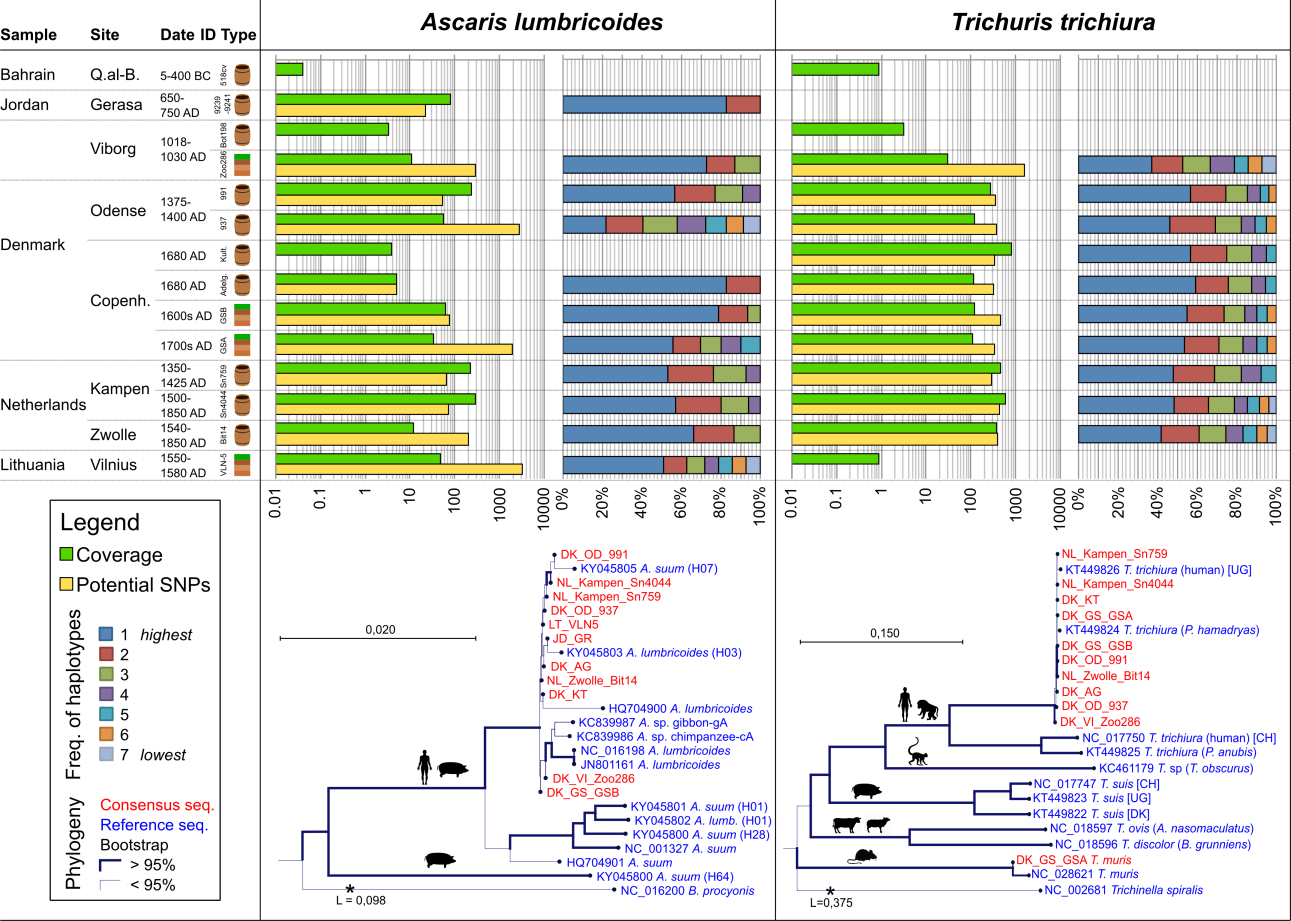
Sequence depth, genetic variation and phylogeny of *Ascaris lumbricoides* and *Trichuris trichiura* mitochondrial genomes. Top left: Depth of coverage and number of potential SNPs when mapping unique hits (Fig 2) to the closest related mitochondrial genomes of Ascaris lumbricoides (KY045805) and Trichuris trichiura (KT449826). Top right: Frequency of identified haplotypes. Average depth coverage of minimally 5–10x was required to estimate the number of potential SNPs and the frequency of haplotypes. Bottom: Phylogenies of consensus mitochondrial genomes and all published mitochondrial genomes. The *T*. *muris* consensus sequence was generated by remapping unique hits to NC_028621. The scale bar indicates number of base substitutions per site.

Plastid sequence hits are reported at the genus level, as the database in its current form is lacking many species. Genus level hits as shown in Fig 3, are collapsed as follows: *Vitis* contains reads assigned to *Vitis* genus and *Vitis vinifera* and *Vitis rotundifolia* species; *Humulus* contains *Humulus lupulus*; *Prunus* contains *Prunus* and *Prunus maximowiczii*; *Pyrus* contains *Pyrus* and *Pyrus spinosa*; *Pisum* contains *Pisum sativum*; *Fagopyrum* contains *Fagopyrum* and *Fagopyrum esculentum*; *Rheum* contains *Rheum palpatum*; *Lupinus* contains *Lupinus luteus*; *Lotus* contains *Lotus japonicus*; *Vaccinium* contains *Vaccinum macrocarpon*; *Hordeum* contains *Hordeum* and *Hordeum vulgare*. Species with reads mapping to more than 1 of 4 extraction controls and more than 2 hits in a single control are not reported in Fig 4. Full lists of helminth, vertebrate and plastids hits are reported in S3, S4 and S6 Tables.

### Mitochondrial genome analysis

Reads assigned to the species *T*. *trichiura* and *T*. *muris* and the *Ascaris* genus by the LCA algorithm, were extracted using *seqtk* version 1.0-r31 and the *subseq* command. Reads were re-mapped to the *T*. *trichiura* sequence from humans in Uganda (KT449826), the *Ascaris* sp. sequence from haplotype 07 (PUG3-1-H07) or the *T*. *muris* sequence (NC_028621) using bowtie2. Sam output was converted to bam format, merged to library level and sorted using command *sort* in *samtools* version 1.2 [28]. Alignment coverage and depths were calculated using Paleomix version 1.1.1, commands *coverage* and *depths* [29]. Mitochondrial consensus sequences were called using the commands *samtools mpileup*, *bcftools call* (version 1.2) and *vcfutils vcf2fq*.

The consensus sequences from *T*. *trichiura* and *T*. *muris* were aligned together with all mitochondrial genome sequence of *Trichuris* genera publicly available at NCBI, as was done for the *Ascaris* genus sequences. Certain reference sequences were re-arranged to start with the *cox*1 gene. Alignment was performed with *mafft* v7[30], using the *globalpair* setting at 1000 iterations. A tree was built through 1000 bootstraps using the Neighbor Joining method and Jukes-Cantor nucleotide distance model in the CLC Sequence Viewer 7 program, which was also used for visualization. The consensus mitochondrial genome sequences were annotated using Mitos WebServer (http://mitos.bioinf.uni-leipzig.de/index.py) and made publicly available (Genbank accession number will be inserted here).

### DNA damage

Ancient DNA damage patterns were assessed using mapDamage v2 [31], for helminth, vertebrate and plastid identified hits. The analyses were performed as an average of all reads assigned accordingly (Fig 2 and S2–S6 Tables). For each library the 5’ C to T transition percentage and the 3’ G to A transition percentage were calculated and the average damage percentages are reported at the sample level.

### Characterization of haplotypes

In order to identify potential SNPs, heterozygous bases were identified from pileup files of each mitochondrial genome assembly. Values for consensus coverage depend on the DNA sequencing error rate and the number of heterozygous bases of the genome analysed. However, as the number of heterozygous bases is markedly lower than the number of homozygous bases, the vast majority of data points will fall around the DNA sequencing error rate. Hence, with an error rate of 5% (sequencing and ancient DNA damage), values for consensus coverage for each position forms a distributions around the average of 95%. To distinguish potential SNPs from the background of the error rate, the average and spread of the consensus coverage data was calculated across each mitochondrial genome and only positions where the consensus coverage could be identified as outliers, *i*.*e*. values outside the main body of the data (<6* *inter quartile range* (IQR)) were used for the analysis. This threshold was also used to distinguish 'true haplotypes' from haplotypes that could be an effect of sequencing errors below.

Next, clusters of potential SNPs were characterized using a window size of the read length (100bp). Sam files were then parsed with respect to the SNP clusters identified: for each cluster, reads covering all potential SNPs of the given cluster were analysed, counting all different haplotypes at that particular SNP cluster. In order to avoid a potential bias from ancient DNA damage, the following bases did not count towards the final haplotype assessment: Thymidine bases within the first ten base pairs of a read where the consensus base was Cytosine and Adenine bases within the last ten bases of a read where the consensus base was Guanine.

To retain only high confidence clusters, clusters were excluded if they were covered by less than 10 reads or less than one fourth of the mean coverage of the mitochondrial genome assembly. Furthermore, to filter out haplotypes that could be a result of sequencing errors, low abundance haplotypes constituting less than the threshold percentage described above (<1–6*IQR) were discarded. After filtering, the estimated (minimum) number of haplotypes for each mitochondrial genome was characterized, defined as the highest number of haplotypes identified in at least two different clusters. The fraction of each haplotype presented in Fig 4 was calculated as the mean fractions across the clusters where the estimated number of haplotypes where identified. This approach was benchmarked using shotgun sequencing data generated from single, modern *T*. *trichiura* worm isolates which all yield a single haplotype (data not shown).

## Results

Samples apparently rich in organic material were collected from recent archaeological excavations and museum collections (S1 Appendix). Samples were screened for the presence of *Ascaris* spp. and *Trichuris* spp. eggs, typically associated with human faeces, of which 21 subsamples from 14 distinct locations were chosen for further processing (Fig 1). The majority of samples were characterized as latrines or cesspits (10/14) and the remaining as deposits or fill layers (4/14). Subsamples were concentrated for 22–35 and 35–100 μm particles by wet sieving; from which 1/10^th^ was subjected to traditional morphological assessment of parasite eggs whereas the remaining material was used to generate 40 blunt-end DNA libraries. Shotgun sequencing generated a total of 1,062,232,288 DNA reads, which after rigorous pre-processing by removing adapter sequences, low complexity reads and PCR duplicates, yielded 839,575,890 reads (details in S1 and S2 Tables). Pre-processed reads were mapped against a mitochondrial reference database and assigned to the lowest common ancestor. After removal of hits to common laboratory contaminant in particular *Homo sapiens* and related primates as well as cattle (*Bos*), chicken (*Gallus*) and pig (*Sus*), which were present in most samples and in some controls, a dataset was compiled and used in the subsequent presentation. This dataset contains 605,635 unique hits (721 hits per million sequenced reads) to helminths (phylum *Nematoda* and *Platyhelminthes*) and 549 unique hits (0.6 per million) to vertebrates as well as 28,141 unique hits to edible plants when mapped against a plastid reference database (33 per million).

### Helminths, vertebrates and edible plants

Taxa assignments, as identified by morphological examination and DNA assignation, were initially compared for individual particle size fractions. In general, parasites which excrete large eggs such as *A*. *lumbricoides*, *Fasciola hepatica*, *Parascaris univalens*, *Toxocara* spp., were identified in 35–100 μm particle size fractions, whereas parasites excreting smaller eggs, *i*.*e*. *Dicrocoelium dendriticum*, *Enterobius vermicularis*, *Taenia* spp. and *Trichuris* spp., were mainly identified in the 22–35 μm particle fractions. However, few discrepancies were noted, most often identification of parasites with small eggs among the larger particles (35–100 μm fraction), resulting from insufficient washing of filters.

Taxa assignments from different particle size fractions were then merged for the 14 samples for which the most abundant taxa are presented in Fig 2 (for complete lists see S3 and S4 Tables). Morphological examination identified 7 different helminth genera while DNA analysis identified 8 genera including 11 distinct species. DNA identified 78% of the morphologically identified taxa (32 of 41), with the 9 taxa missed by DNA being: *Capillaria* (1 sample, no reference sequence available), *Diphyllobothrium* (2), *Enterobius* (1), *Taenia* (3) and *Toxocara* (2). In contrast, DNA provided species level identifications for 6 different genera missed by morphological identification: *Dicrocoelium dendriticum* (6 samples), *Diphyllobothrium latum* (1), *Enterobius vermicularis* (2), *Fasciola hepatica* (1), *Parascaris univalens* (2) and *Taenia hydatigena* (1), and additionally provided species assignation for two distinct *Taenia* species (*T*. *solium* and *T*. *hydatigena*) and three distinct *Trichuris* species (*T*. *trichiura*, *T*. *suis* and *T*. *muris*). DNA from both helminth and it’s typical definitive host were identified in a number of cases: *D*. *dendriticum* and *Ovis* (sheep) (identified in 4 of 5 cases), *F*. *hepatica* and *Ovis* (sheep) (1/1), *P*. *univalens* and *Equus* (horse) (1/2), *Taenia hydatigena* and *Canis* (dog) (1/3), *T*. *muris* and rodents (*Rattus rattus* (Black rat)) (1/5). Ancient DNA damage profiles are shown for helminth identified reads (Fig 2). Damage profiles for vertebrate identified reads could not be determined due to the very low number of reads.

The most abundant edible plants show a large diversity of taxa present in samples from the Northern European areas (Fig 3 and S6 Table). Identified plant taxa exhibit ancient DNA damage profiles that are comparable with those presented for the helminth findings, although lower rates were observed for the two youngest samples from the Netherlands (Kamper 4044 and Zwolle) (Fig 2.).

### Genetic diversity and phylogenetic relationships

Reads assigned to *A*. *lumbricoides*, *T*. *muris* and *T*. *trichiura* were extracted and remapped (as the database contained multiple genomes of each species) to their closest reference mitochondrial genomes for further analysis. This yielded 11 samples with more than 90% bases covered for *A*. *lumbricoides* (average 96.6%), 10 samples for *T*. *trichiura* (average 100%) and one sample for *T*. *muris* (98.4%) (Fig 4, S3 and S4 Tables). Average read depth of coverage was 96x for *A*. *lumbricoides* genomes (range 5x-284x), 303x for *T*. *trichiura* genomes (range 30x-818x) and 7x for *T*. *muris*. In particular *A*. *lumbricoides* genome coverage was low in areas of low sequence complexity (*i*.*e*. immediately up/downstream of bp 2860, 6420, 6700, 7500, 7800, 12020). This is possibly a result of: 1) the rigorous pre-processing of sequence data or 2) that these regions are conserved between closely related species, resulting in read assignation to family level or higher. In contrast, the *T*. *trichiura* mitochondrial genome coverage only exhibited one problematic region (*i*.*e*. AT repeats up/downstream of bp 3230).

The number of potential SNPs, identified as positions with low consensus coverage, was estimated for all reconstructed mitochondrial genomes. *A*. *lumbricoides* sequences exhibited some very high (Lithuania—VLN5 = 3205) and very low (Denmark, Gammel Strand B = 5, Jordan Gerasa = 22) estimated values, while averaging 792 potential SNPs across all samples. In contrast, the number of potential SNPs in *T*. *trichiura* sequences ranged 297–457 when excluding the Zoo286 sample from Viborg, Denmark (1605 SNPs), averaging 492 potential SNPs across all samples.

The number of potential haplotypes was then counted in all samples, based on clusters of potential SNPs separated by no more than 100 bp (read length). This provides a conservative estimate, as it is restricted to haplotypes of SNPs spanned by a single sequencing read. Various thresholds were implemented to distinguish 'true haplotypes' from those originating from potential sequencing errors or DNA damage. Frequencies of haplotypes were estimated, with *T*. *trichiura* exhibiting a more uniform distribution than *A*. *lumbricoides*, as well as a higher number of identified haplotypes 6.0 (ranging 5–7) compared to 4.0 (ranging 2–7) for *A*. *lumbricoides*. To rule out that the estimated number of potential SNPs and haplotypes were influenced by the number of eggs in the sample or the sequencing depth, the following linear regression analysis were performed showing no statistical correlations: the number of eggs and number of potential SNPs in a sample (R^2^ = 0.11 –*A*. *lumbricoides*; 0.02—*T*. *trichiura*), the number of sequencing reads and number of potential SNPs (R^2^ = 0.41—*A*. *lumbricoides*; 0.04—*T*. *trichiura*), the number of potential SNPs and average depth of coverage (R^2^ = 0.10 –*A*. *lumbricoides*; 0.14—*T*. *trichiura*) and the number of haplotypes and average depth of coverage (R^2^ = 0.003 –*A*. *lumbricoides*; 0.04 –*T*. *trichiura*).

Individual haplotype sequences could not be called as a result of the short read length; hence consensus sequences were called to investigate phylogenetic affiliations (Fig 4). *A*. *lumbricoides* genomes group together with haplotypes *H03* and *H07* in a cluster which elsewhere is designated cluster B[10]. *T*. *trichiura* sequences grouped with reference sequence from human isolates from Uganda, distinctly different to the other *T*. *trichiura* cluster observed in humans from China.

## Discussion

Using a novel approach of applying shotgun sequencing on ancient parasite eggs that have been purified by filtering, we have obtained a new and much more detailed insight into parasitic infections of human populations of the past. In addition, the complementary profiling of helminth, vertebrate and plant DNA proved highly informative in the study of ancient health, human-animal interactions as well as the animals and plants they exploited. Finally, the reconstruction of full mitochondrial parasite genomes from *Ascaris lumbricoides*, *Trichuris trichiura* and *Trichuris muris*, elucidated the genetic diversity and provided insights into epidemiology and parasite biology.

We identified helminths which are directly transmitted between humans (*A*. *lumbricoides*, *E*. *vermicularis* and *T*. *trichiura*) by faecal contamination of the immediate environment, but also human helminths with indirect transmission where fish (*D*. *latum*) and pig (*T*. *solium*) are required as intermediate host, indicating human consumption of raw or undercooked fish and pork. The identification of parasites for which humans are not the definitive host e.g. *D*. *dendriticum* (sheep definitive host), *F*. *hepatica* (sheep), *P*. *univalens* (horse), *T*. *hydatigena* (dog), *T*. *muris* (rodent) and *T*. *suis* (pig) indicates that faeces from these domestic and synantropic animals are present in the samples and that they were living in proximity to humans at the respective sites. Notably, we identified two species of *Trichuris* that do not infect humans, *T*. *suis* from pigs and *T*. *muris* from rodents, which most likely would have been wrongly assigned to *T*. *trichiura* using traditional morphological approaches [1,14,15]. Similarly, DNA identification of *T*. *hydatigena* eggs is interesting as it reveals the presence of a canid definitive host. Using classical methodology, such eggs would very likely have been assigned to human infection with *T*. *solium*, as also found in the present study, or *T*. *saginata* after consumption of pork or beef, respectively [14,15]. Microscopy did identify *Capillaria* eggs (not in reference database) in one sample and *Toxocara* eggs in two samples that were not identified in the DNA analysis.

We acknowledge that the taxonomic status of *A*. *lumbricoides* and *A*. *suum* is contentious as close similarities in mitochondrial and single nuclear markers have been identified in isolates from pigs and humans, including shared genotypes and haplotypes [10,32]. In spite of this, we are confident that the reads assigned to *A*. *lumbricoides* in this study stems almost solely from human *Ascaris* infections due to co-detection with the highly host specific *T*. *trichiura*. However, *T*. *suis* was identified in two samples (54 reads in sample 937 from Odense, Denmark and 6 reads in the Lithuania VLN-5 sample) indicating that pig faeces were present in these samples. Assuming co-detection of *T*. *suis* and *A*. *suum* in pigs, similar to the level observed for *T*. *trichiura* and *A*. *lumbricoides* in humans, would imply that *A*. *suum* was also be present at those sites and in those samples. This may explain why these two samples exhibited the highest number of potential SNPs in their consensus mitochondrial sequences.

Reconstructing complete mitochondrial genomes of ancient parasites is novel and analysis of the associated genetic variation, opens for a more detailed level of information regarding the transmission biology of parasites (Fig 4). As the sequencing data stems from many different eggs, originating from many different worms and potentially many different hosts, the reconstructed mitochondrial genomes should be considered consensus sequences of the population. Doing so, we identified a higher number of haplotypes in *T*. *trichiura* (average of 6.0 across samples) compared to *A*. *lumbricoides* (4.0). We interpret this as a result of worm biology, particularly in relation to the reproductive potential of an individual worm. The effective worm population of an infection, hence the worm burden, is typically 1–15 worms for *Ascaris* infections [33] and 10–60 worms for *Trichuris* infections [34]. The larger worm burden provides a larger gene pool in *T*. *trichiura* infections and may explain the higher number of haplotypes.

Despite that the mitochondrial genome sequence of *A*. *lumbricoides* cannot be used to distinguish *Ascaris* from humans and pigs, we show mitochondrial sequence affiliation to cluster B for all samples, particularly close resemblance to haplotype 03 and 07. Although, H03 and H07 are identified among isolates from humans and pigs in Africa, South America, and Asia they are more frequently found among samples from China [10,35]. In contrast, haplotype H01 belonging to cluster A is the most common type among samples form humans in Africa [10]. This suggest that *A*. *lumbricoides* from Northern Europe and Jordan (650–1700AD) are more closely related to worms in Asia than to worms in Africa, which is in contrast to what we see for *T*. *trichiura* as outlined below. However, comparison of frequencies of haplotypes between geographical regions has to be interpreted with some caution due to the low number of genomes generated in the current study and as the phylogeny of *Ascaris* based on mitochondrial DNA is complex (Fig 4).

In sheer contrast, the *Trichuris* sp. mitochondrial sequences can provide identification to species level and has recently been used to address phylogeographic distribution of *T*. *trichiura* and *T*. *suis* [9,36]. The identified phylogeny suggests that Northern European *T*. *trichiura* (1000–1700 AD) was very closely related to worms found in present day Uganda while it has significantly diverged from present day China. This surprising finding indicates very high degrees of genetic mixing between *T*. *trichiura* in Uganda and Northern Europe (1000–1700AD) which does not fully align with the hypothesis that *T*. *trichiura* was dispersed globally along with human migrations [8,9]. Further, the clear segregation between isolates from Africa+Europe versus China+Americas indicates that potentially two subspecies of *T*. *trichiura* should be considered.

Identification of DNA in modern faecal samples has previously been used as indicators of host diet [37,38]. Vertebrate DNA in ancient latrines thus reflects either the diet of the people who used it for defecation, or concurrent deposition of animal remains or excrements. The vertebrate DNA findings here could not be authenticated through ancient DNA damage profiles due to a very few number of reads. However, authentication was obtained by inclusion of negative controls, which in no instances resulted in assignment of reads to the positively identified taxa. Furthermore, the presented vertebrate findings are sound based on historical contexts, giving validity to the use of shotgun metagenomic approaches.

In particular, we show that vertebrate DNA from domesticated animals is more abundant compared to that from wildlife animals. Wildlife findings are interesting as they clearly indicate hunting and fishing practices, as shown in samples from Denmark (1018–1400 AD), where *Baleaenoptera physalus* (finwhale), *Capreolus capreolus* (roedeer) and *Lepus europaeus* (European hare) were identified. Large whales, including fin whales, are still recurrently stranding on beaches in Denmark, which would have provided easy access to large amounts of meat and fat, while catching live fin whales would have been a major accomplishment in the Viking-age. A comparable exploitation of dead whale by ancient cultures in the Arctic was recently suggested by us [16]. Another example of consumption of marine mammals, although below threshold for inclusion in Fig 2, is identification of harbour porpoise DNA in a latrine sample from Odense, Denmark (1375–1400 AD), a species that has been exploited locally and is still native to Danish waters. Five fish species were identified in samples from Kampen in the Netherlands (1350–1850 AD) and Copenhagen, Denmark (1600s AD). Only saltwater fish species were identified in Copenhagen (*Clupea harengus* (Atlantic herring) and *Gadus morhua* (Atlantic cod)), while both fresh- (*Abramis brama* (common bream) and *Anguilla anguilla* (European eel)) and saltwater species (*G*. *morhua* and *Hippoglossus hippoglossus* (Atlantic halibut)) were identified in Kampen. This corresponds with their respective locations, Copenhagen at the sea and Kampen on the banks of the river Ijssel, just upstream of its estuary.

Earlier studies have used ancient environmental DNA analysis to study past ecosystems and human activities [16,39–42]. The plant material found in latrines and deposits in the present study should mainly be considered a result of human deposition, through faecal excrements, general waste management (*e*.*g*. kitchen refuse and as part of animal faeces) or as a means to initiate biological degradation processes in the latrine (*e*.*g*. leaves, ferns), but only minimally as a result of natural deposition. The most abundant edible plants found in the samples from Northern Europe included cabbages (*Brassica*), buckwheat (*Fagopyrum*), wheat (*Hordeum*), sweet peas (*Lathyrus*), legumes (*Lotus*), peas (*Pisum*) and more seasonal foods herein mainly fruits: strawberries (*Fragaria*), plums/cherries (*Prunus*), pears (*Pyrus*), rhubarb (*Rheum*), and berries possibly cranberry/blueberry (*Vaccinium*). Notably, carrots and potatoes which are consumed in large quantities today, were not identified, in agreement with their gradual introduction in Northern Europe from the 16–1700s [43,44]. The present study did not allow for a detailed analysis of geographic preferences for the above mentioned plants, but grapes (*Vitis*), as often used in wine production, were identified in high concentration in the Netherlands and less so in Copenhagen, Denmark. In contrast, hop (*Humulus*), as often used in beer production, was identified in Odense and Copenhagen, Denmark and in Lithuania. Finally, tobacco (*Nicotiana*) was identified at the docks in Copenhagen (Gammel Strand), which was also the site of highest sample diversity, plant as well as helminth and vertebrate.

In conclusion, sequencing of ancient samples often involves a DNA target enrichment step in order to obtain sufficient concentrations of endogenous DNA for sequencing [45]. As a novel approach we have combined pre-concentration of parasite eggs with shotgun sequencing to conduct metagenomic studies and to reconstruct mitochondrial genomes. In addition, the approach employed has an obvious potential for reconstructing nuclear genomes of ancient parasites.

## Supporting information

S1 Appendix Description of sampling locations. (DOCX)

S1 Table Sequencing libraries information. (XLSX)

S2 Table Sample level information. (XLSX)

S3 Table Sequencing reads assigned to helminths. (XLSX)

S4 Table Sequencing reads assigned to vertebrates. (XLSX)

S5 Table Mitochondrial genome coverage and damage. (XLSX)

S6 Table Sequencing read assignment using plastid database. (XLSX)
