# Supplementary material for: Ancient DNA from latrines in Northern Europe and the Middle East (500 BC–1700 AD) reveals past parasites and diet
Source: PLoS One. 2018 Apr 25;13(4):e0195481. doi: 10.1371/journal.pone.0195481 (PMC5918799; doi:10.1371/journal.pone.0195481)
Supplement: S1 Appendix — (DOCX) [file pone.0195481.s001.docx]

**Ancient DNA from latrines in Northern Europe and the Middle East (500 BC – 1700 AD) reveals past parasites and diet**

Martin Jensen Søe, Peter Nejsum, Frederik Valeur Seersholm, Brian Lund Fredensborg, Ruben Habraken, Kirstine Haase, Mette Marie Hald, Rikke Simonsen, Flemming Højlund, Louise Blanke, Inga Merkyte, Eske Willerslev, Christian Moliin Outzen Kapel.

**S1 Appendix**

**Sample locations**

Bahrain, 5-400 BC

The city of Qala’at al-Bahrain, situated on the northcoast of Bahrain, seems especially wealthy in the Achaemenian period. In three different excavations with an internal distance of 1-200 m the same type of urban architecture has been uncovered, with a variety of different-sized buildings, often constructed in finely cut limestone ashlars. Each building normally had one lavatory, equipped with two or, in rare cases, three toilets. The toilets were installed in low, plastered platforms with shield-shaped openings down into more than 1 m deep subterranean plastered tanks. The sample (518.CV) derives from such a tank in a three-toilet lavatory in Excavation 518, and it was excavated in 1958 by Poul Kjærum from Moesgaard Museum^1^.

Jordan, 650-750 AD

The ancient city of Gerasa is located in Northern Jordan, some 35 km from the modern capital Amman. The city was founded in the Hellenistic period and was in use well into the Islamic period. The samples derive from a latrine in the Central Bathhouse, located in the commercial centre of Gerasa in the intersection between two of the city’s main thoroughfares. The bathhouse is located adjacent to shops and to several larger communal buildings. Therefore, it is believed that the general public frequented the bathhouse and associated latrine and that the samples are representative of the average population. Ceramic potsherds and coins found within the deposit suggest that the samples date to the later uses of the latrine, immediately before the building was closed down in the late seventh or early eighth century CE.

The excavation of the Central Bathhouse was carried out between 2002 and 2010 as a part of the Danish-Jordanian Islamic Jarash Project under the direction of Alan Walmsley, University of Copenhagen. The project is most grateful to the Jordanian Department of Antiquities, Amman and Jarash offices, for their invaluable support and collaboration.

Samples were collected by Louise Blanke in 2010.

Denmark, Viborg, 1018-1030 AD

Samples were collected by Allan Roepstorff in 2001 and have been stored at the Royal Veterinary and Agricultural University in Copenhagen (now University of Copenhagen). Samples are from a latrine and waste-dumping pit situated adjacent to a workshop in a Viking-age village located at the present day city of Viborg, Denmark. The workshop and it’s immediate surroundings were excavated and archaeological findings as well as morphological assessments of parasite eggs have previously been described^2^. The examined contexts were dendrochronologically dated to 1018-1030 AD.

Ancient DNA based analysis of the parasite eggs isolated from these contexts have previously been presented using amplicon based approaches^3^. Two samples contained significantly higher concentrations of *Trichuris* sp. eggs than others and these were included in the present study:

- The Bot198 sample, which was identified as a waste-dumping pit. This pit was also shown to contain human faecal material and hence was thought to serve as latrine in times when the designated latrine (Zoo286) was full.
- The Zoo286 sample from a pit designated as latrine.

Denmark, Odense, 1375-1400 AD

Samples from barrels at OBM 9776, I. Vilhelm Wernes Plads, excavated during campaign from May 2013 to September 2014.

Odense is now the third largest city in Denmark and is located in the island of Funen. Odense is first mentioned in the written sources in 988 CE and a ring fortress from the 10^th^ Century indicates that Odense is a place of significance. From the 12^th^ and 13^th^ Century there is no doubt that Odense is a growing town. In the 14^th^ Century the town plots are densely build and the present samples derive from a latrine from this period.

The latrine consists of two barrels dug into the ground. Remains of posts indicate that a small building covered it. This building was leaning against the back of a house with a large kitchen placed centrally on the plot. The latrine seems to only serve this specific household.

The town plot was located in the centre of Odense adjacent to the main street leading across Funen. This part of the town was probably inhabited by wealthy and influential townspeople. The macrofossils from the latrine are currently being analysed and this – along with the present analysis – will hopefully tell us more about the inhabitants of 14^th^ Century Odense.

Four samples from two barrels 991 (X2172, X2173) and 937 (X2219, X2220) were analysed. The barrels are dendrochronologically dated to 1348-1358 CE. They are reused barrels, most probably initially used for storing salt, from Poland and the stratigraphy and finds indicate a date around 1375-1400 CE.

Excavation leaders, Odense City Museums: Curator Jakob Tue Christensen, Archaeologist Kirstine Haase. Dendrochronology: Aoife Daly, Ph.D., dendro.dk.


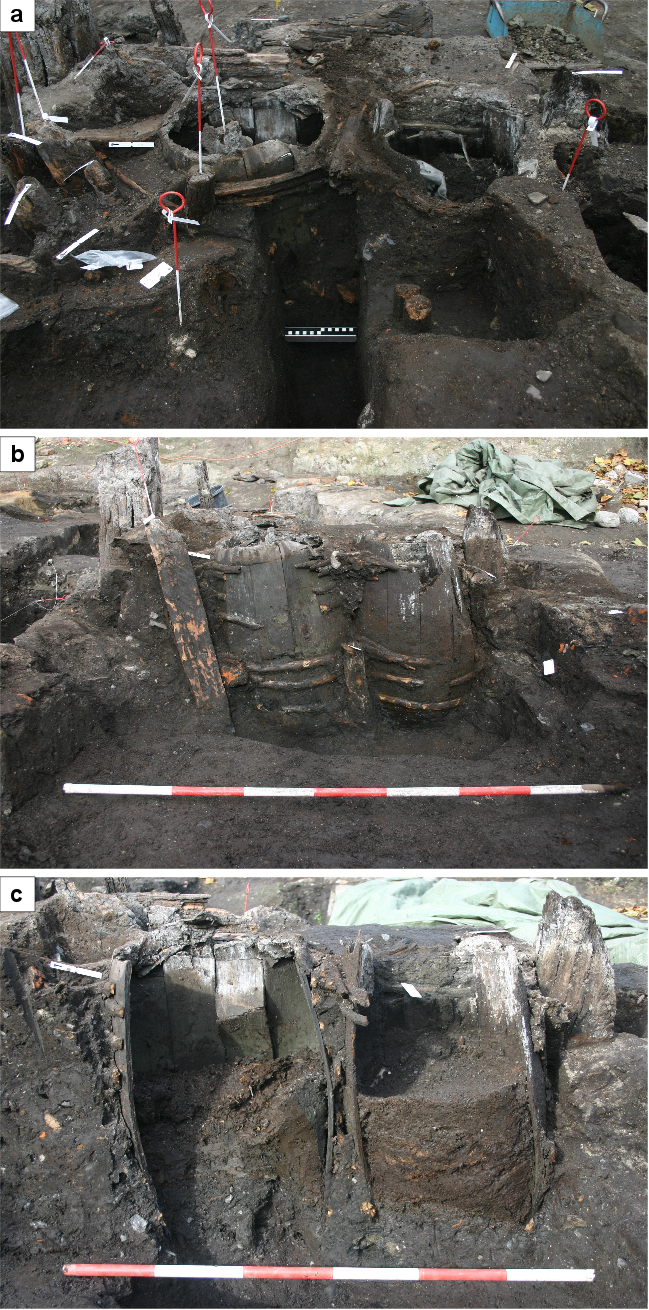


Figure 1 | Latrine barrels (lag 991 and lag 937) from Odense, Denmark (1375-1400 AD). Three images show the progress of excavating and opening the barrels. The samples analyzed here were collected from the top and bottom of both barrels. Pictures from Odense City Museums.

Denmark, Copenhagen, Gammel Strand, 1600s AD

Samples were collected as part of an ongoing archaeological excavation at Metro station building site in 2014. Gammel Strand is situated in the very centre of historical Copenhagen at the site of the natural harbour. A proper harbour was established over several hundred years (16^th^ – 18^th^ century) by developing wharfs through depositing refuse in wooden frames. Development of the wharf was done in sections for which samples used in this study were taken from those developed in the 17^th^ century. Samples from the same GPS position should be considered contemporary.

**GSA2:** Taken from 17^th^ century wharf development (GSA), 120 cm over sand bottom.

**GSA5:** 17^th^ century, taken at 40 cm over sand bottom.

**GSB2:** Taken from 17^th^ century wharf development (GSB), 90 cm over sand bottom.

**GSB4:** 17^th^ century, taken at 20 cm over sand bottom.

Denmark, Copenhagen, Adelgade, 1680 AD

In 2013 an archaeological excavation was undertaken prior to the construction of a new office building in Adelgade 12 close to the old centre of Copenhagen.

The excavation revealed more than 400 years of coherent cultural history of the site. A central element in the excavation was an old road preserved in several phases from the Middle Ages until the 1990s. In the medieval period the road was lying outside Copenhagen leading from the Eastgate of the city to Elsinore. On one side of the road part of an old moat was excavated which was established by King Christian 4^th^ in the beginning of the 1600s as a reinforcement of the city’s defences. On the other side of the road was a big estate with nice gardens belonging to the king’s doctor who like many other wealthy citizens also had a house in the countryside outside the city to enjoy the privilege of light and fresh air.

In the mid 1600s the fortification of the city was moved further out and the area came to lie inside the city boundaries. Upcoming industries moved to the more spacious area and during the next hundreds of years the number of people and buildings in the street was increasing. The excavated remains of the houses built on the filled-in moat and ditches of the gardens showed the poor quality of what became one of the most deprived neighbourhoods in Copenhagen with prostitution, bars etc. In the long narrow backyards of the 1700-1800s barrels and waste pits from local brandy production were found along with wooden water pipes transporting drinking water.

The wooden water pipes were replacing the older wells in the city that were known for their poor water quality. In one of the backyards an abandoned well was found. It was built from pine planks, dating to between c. 1650 and 1750. The deposit in the well case suggests it had a possibly secondary usage as cesspit. A plant macrofossil analysis of this deposit reveals the diet presumably of the people living on the plot showing a mix of edible species, e.g. black mustard and berries (raspberry/blackberry and blackthorn), and common field weeds like corn-cockle growing in rye fields.

Gareth Dickinson collected the analysed sample, from the well, on the 9^th^ of August 2013.

Figure 3 | Well case at Adelgade, Copenhagen from which the sample was collected. Picture from Museum of Copenhagen.

Denmark, Copenhagen, Kultorvet, 1680s AD

Kultorvet square is situated in central Copenhagen. The square was established as a consequence of a devastating fire, which raged for three days through the western and north-western parts of Copenhagen in the autumn of 1728, destroying large parts of the Renaissance city. Before the fire, the Kultorvet area had contained a block of houses used for light industry and residential buildings. Afterwards, the buildings on Kultorvet where not rebuilt, but instead the area was levelled and turned into a square, essentially sealing the burned-out houses below.

Excavations in 2011 revealed a range of archaeological features including houses mainly built in Dutch Renaissance style, garden plots, sheds, baking ovens, basements, pavements, roads and outdoor latrines. The artefacts recovered included domestic and imported ceramics and glass along with organic and metallic artefacts and tools.

The latrines, one of which was sampled for this report, were found under a road that had been constructed shortly before 1690. They were located inside a backyard of one of the houses in the area. The remains of wooden posts that had formed a construction around the latrines were found around the buried barrels, indicating a hasty demolition of the buildings as the road was built. The latrines consisted of two large barrels that had been placed next to each other. Both had been dug into the ground. They where two-thirds filled with human faeces and, apart from a clear division of light (straw) and heavy (brick fragments) components, there was no visible stratigraphy in the barrels, possibly indicating a frequent filling and emptying of the barrels.

Samples were collected by Mette Marie Hald on the 28^th^ of April 2011 and have subsequently undergone archaeobotanical analysis by Hald.

Sample ID: KBM 3959 - PM1176 (SD1173) and KBM 3959 - PM1177 (SD1174).


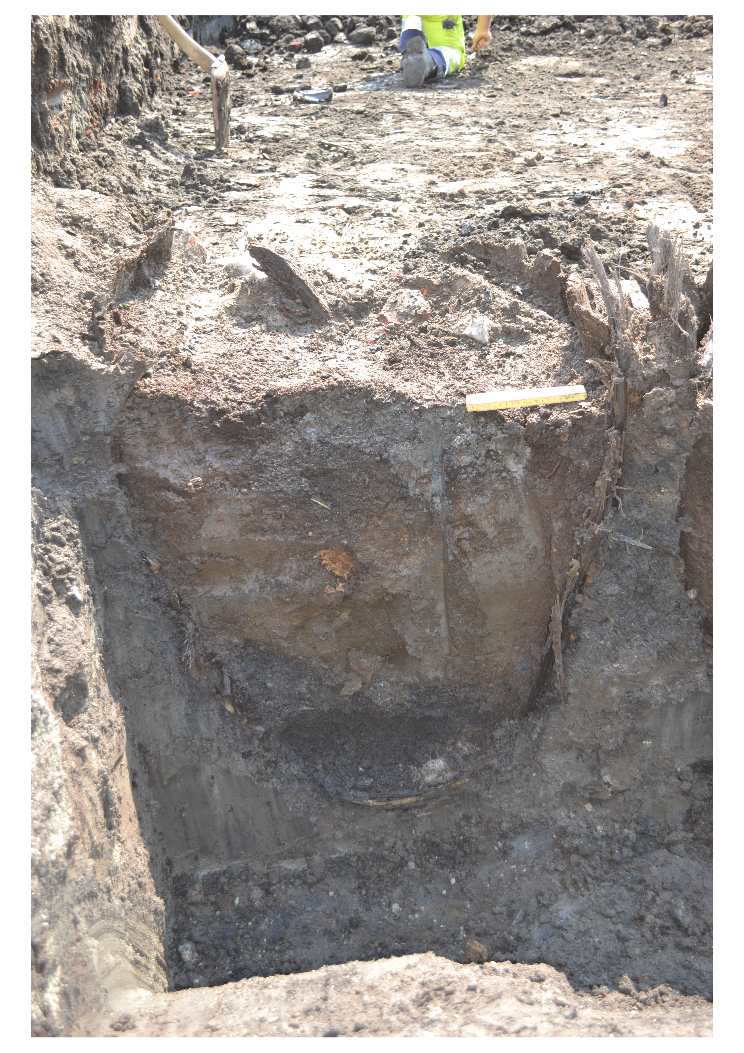


Figure 2 | Latrine barrel from Kultorvet, Copenhagen, Denmark. Picture from Museum of Copenhagen.

The Netherlands, Kampen, 1350-1850 AD

Samples are from a medieval guesthouse in the Hansa city of Kampen. Historic data shows that the site opened in the second half of the 14th century. Before the opening there were a couple of wooden houses located on the site dating to the early 14th century, these have been dated by the bricks from the floor foundation, and historic data. When it was opened in 1382, the guesthouse harboured the sick and travellers. Around 1460 they started to harbour the old as well until it closed in the 19^th^ century^4^. The site was excavated in two faces. During both excavations dung pits, barrel pits, and cesspits were recovered which are to be believed from before the opening of the guesthouse and during the time it was open. The two samples analysed in this study were taken from the second excavation. Research is currently conducted at this site by the city archaeologists of Zwolle. Samples date to between the second half of the 14^th^ century and 1850, of which the wooden pits are from the earlier period.

**Sn 759** (find 525) was taken from a wooden barrel pit on the site. This barrel pit is located near one of the wooden houses dating from before the opening of the guesthouse. Due to its location it is assumed to belong to one of the wooden house constructed before the guesthouse, but which may still have been in use at the time the guesthouse was opened. Dating is set to the second half of the 14^th^ century until early 15^th^ century.

**Sn 4044** (find 272) was taken from a brick cesspit. This cesspit was one of the cesspits lodged in the wall of the guesthouse. Dating is based on the type of bricks from which the cesspit was constructed and set at 1500-1850 AD.

The Netherlands, Zwolle, 1540-1850 AD

**The Bit14** sample was taken from a cess cellar of a nobleman’s house from Zwolle. The cess cellar was located on the premises against the back wall of the house at the Nieuwstraat. Pottery found in the cesspit was used in the dating of the cellar. The cellar is dated between 1540-1850, however a hiatus in the pottery assembly was witnessed in the 17^th^ century. It is unknown if the sample dates to the earlier or later than this hiatus.

Lithuania, Vilnius, 1550-1580 AD

The excavation site, Boksto (Tower) Street in central Vilnius, is located at the defence wall constructed 1503-1522 (enclosing an area of 37.18 ha), at one of the city gates, The Savior Gate.

The VLN-5 sample was collected from a trench dated to 1550-1580. The trench constitutes more than 3.5 m of refuse deposits made above erratic sand. Sample VLN-5 was collected from a heterogeneous, organic rich layer containing animal bones and domestic refuse.

The layers were sealed in the 18^th^ century as a building was constructed on top of them.


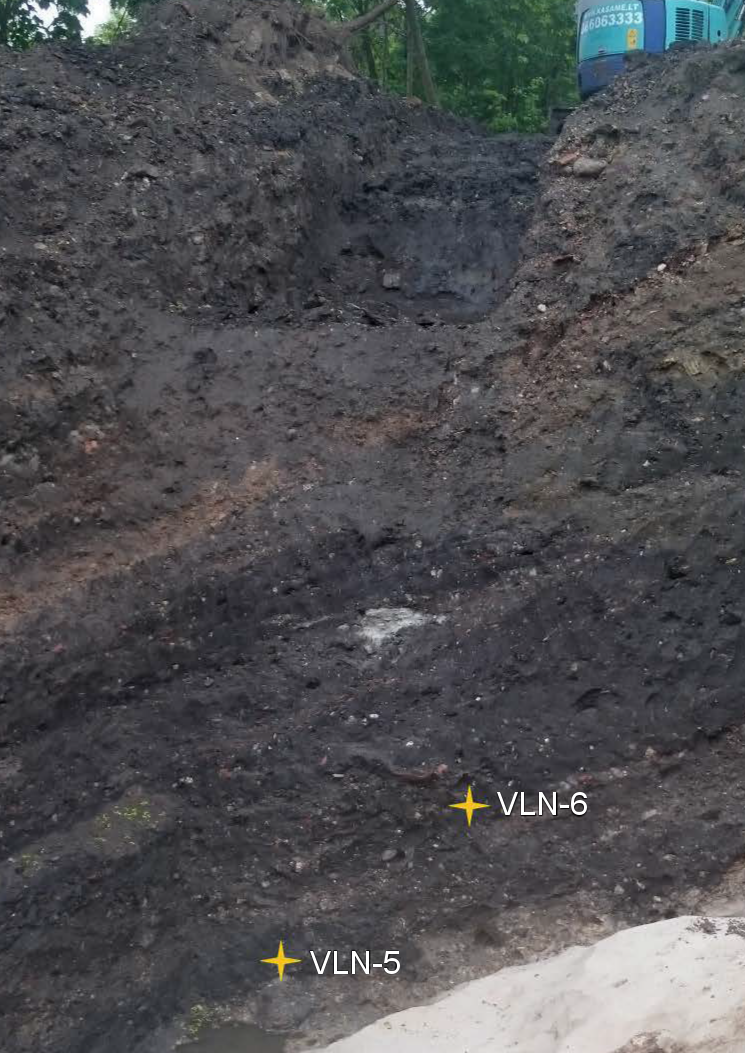


Figure 4 | Profile at Vilnius, Lithuania. Organic rich layer is observed from which the VLN-5 sample was collected. Picture from Inga Merkyte.

**References**

1. Højlund, F. & Andersen, H. H. *Qala´at al-Bahrain, Volume 2, The Central Monumental Buildings*. (Jutland Archaeological Society, 1997).

2. Iversen, M. *Viborg Søndersø 1018-1030, arkæologi og naturvidenskab i et værkstedsområde fra vikingetid*. (Jysk Arkæologisk Selskab and Viborg Stiftsmuseum, 2005).

3. Søe, M. J., Nejsum, P., Fredensborg, B. L. & Kapel, C. M. O. DNA typing of ancient parasite eggs from environmental samples identifies human and animal worm infections in Viking-age settlement. *J. Parasitol.* **101,** 57–63 (2015).

4. Speet, B. M. J. in *Historische stedenatlas van Nederland* (ed. van Herwijnen, G., van de Kieft C., Visser, J.C., Wegner, J. G.) (Delftse Universitaire Pers, 1986).
